# The National Health Service–Galleri multicancer screening trial: explanation and justification of unique and important design issues

**DOI:** 10.1093/jnci/djaf218

**Published:** 2025-08-09

**Authors:** Peter Sasieni, Charles Swanton, Richard D Neal

**Affiliations:** Centre for Cancer Screening, Prevention and Early Diagnosis, Wolfson Institute of Population Health, Queen Mary University of London, London, United Kingdom; Cancer Research UK Lung Cancer Centre of Excellence, University College London Cancer Institute, London, United Kingdom; Cancer Evolution and Genome Instability Laboratory, Francis Crick Institute, London, United Kingdom; Faculty of Health and Life Sciences, Department of Health and Community Sciences, University of Exeter, Exeter, United Kingdom

## Abstract

Despite there being a plethora of multicancer early detection tests, the National Health Service (NHS)–Galleri (ISRCTN91431511) is the only randomized controlled trial (RCT) of a multicancer liquid biopsy in a screening setting thus far. The NHS-Galleri trial has generated much debate, and it has been criticized in the medical press. Some of these criticisms stem from differing opinions over the choice of primary endpoint, others from poor reporting in statements to journalists from those not directly involved in the trial. Some of the debate is positive and relates to the speed of enrollment and the equity in participation, which have shown what is possible in large population-based RCTs. Here we explain our reasoning for undertaking the trial and designing it the way we did. We focus on the reason to consider multicancer screening and why we felt that the results from nonrandomized clinical studies of GRAIL’s Galleri test justified a large RCT. We also consider the slow progress in adopting effective cancer screening historically and in reducing cancer mortality through early detection. There is a need to plan now for future research and implementation depending on the results of the trial. NHS-Galleri is the first double-blind cancer screening RCT. It also, unusually, uses late-stage cancer incidence (rather than cancer mortality) as its primary outcome.

## Introduction

The National Health Service (NHS)–Galleri (ISRCTN91431511) is a randomized controlled trial (RCT) designed to assess the clinical utility of annual screening with GRAIL, Inc’s multicancer early detection test.^1^ The trial commenced in August 2021. It completed recruitment in July 2022 and completed the third round of blood sample collection in July 2024. Participants are now being followed for cancer incidence. Results are expected in 2026.

There have been misunderstandings in the press over the status of a pilot implementation of multicancer early detection test screening by NHS England for which a decision was made not to proceed. The pilot was never part of the NHS-Galleri trial. NHS England had made provision to run a pilot in parallel with the trial (but in different parts of the country) if early results from the trial looked to be extremely promising. There was no planned interim analysis of the trial, but it was agreed that named individuals within NHS England could receive a closed report—ordinarily only shared with the trial’s independent data monitoring committee—with limited unblinded results pertaining to the first-year postrandomization. It should be emphasized that the closed report did not include results on cancer mortality or on the incidence of stage III-IV (ie, stage III or stage IV) cancer, which is the primary endpoint of the trial. Whatever the results from the first year, the trial would have, and did, continue to complete the third round of blood collection (screening) and subsequent follow-up. In the event, the results, to which we as the chief investigators are blinded, were insufficient to justify running a pilot screening program alongside the trial. It is reported that “the first year data showed a high level of accuracy for the test,” but NHS England did not find preliminary results from the first year of the NHS-Galleri trial “compelling enough to justify proceeding straight away with a large-scale pilot program of the test in NHS clinical practice.”^2^ It is our understanding that the 12-month results in terms of a reduction in stage IV cancers needed to be exceptional to trigger the pilot. We think that was never realistic because of the impact of prevalent cancers on the early results in screening trials. For instance, the National Lung Screening Trial, which demonstrated a statistically significant reduction in lung cancer mortality after 3 rounds of annual screening,^3^ did not find any reduction in stage IV cancer in the first round.^4^

## Detailed analysis

### Why multicancer screening?

Cancer is common—approximately half of people in the United Kingdom will be diagnosed with cancer,^5^ and nearly a quarter of deaths are from cancer. For most cancer sites, the difference in fatality from early to late stage is dramatic. Current screening programs target a few types of cancer that cause just 17% of cancer deaths in the United Kingdom. But each screen has a nonnegligible chance of a false-positive result, and most individuals screened regularly for several cancer types will have at least 1 false-positive over their lifetime. Further, the more single cancer screening programs, the less likely that any one individual will participate in all of them. A single multicancer test may identify many types of cancer. Multicancer early detection tests offer great potential for screening, provided false-positives can be controlled. A test detecting 20% of all early stage cancers is likely to have greater benefit than one that finds 80% of a single cancer type. With their emphasis on specificity over sensitivity, however, current multicancer early detection tests would not replace colorectal, breast, or lung screening—rather they would complement existing screening programs.

Blood tests analyzing cell-free DNA (cfDNA) for biomarkers of cancer have received great interest. The larger the tumor burden (macro- and microscopic), the more cfDNA will be shed.^6-8^ Similarly, the greater the cell turnover, proliferation, and underlying genome instability, the more cfDNA will be shed. Thus, the sensitivity of a cfDNA-based test will likely increase with tumor size and aggressiveness.

There are great inequities in cancer control globally both between and within countries. In high-income countries, this often translates to more later-stage cancers in those who are more deprived and often those from ethnic minorities. Because those who are currently diagnosed late have the most to gain from screening, multicancer screening has the potential to reduce inequalities. However, within countries, cancer screening coverage is lowest in those who are most disadvantaged. Thus, ironically, introducing a new cancer screening program could exacerbate inequalities. However, increased health inequalities are by no means inevitable.^9^ A focus on reducing inequalities requires quality management and targeted communication strategies to reach those with the greatest need.

### Why an RCT?

By 2020, GRAIL had evaluated Galleri, a locked test (assay and algorithm), in clinical studies. The test was only rarely (0.5%) positive on bloods from people without cancer but was positive in approximately half (51.5%) of patients (already diagnosed) with a mix of stages and more than 50 cancer types.^10^ Sensitivity increased with stage (17%, 40%, 77%, and 90% for stages I, II, III, and IV, respectively). Stage-specific sensitivities were higher for 12 prespecified cancers responsible for more than 60% of UK cancer deaths (37%, 70%, 87%, and 93%).^11^ Sensitivity for some stage II cancers was higher: 80% for lung, 85% for colorectal, and 65% for esophagus but only 5% for prostate. When positive, Galleri correctly identified the site of origin (on the first attempt) in 88.7% of cancers. In a prospective longitudinal study, PATHFINDER, the original version of the Galleri test led to the diagnosis of 36 cancers in 35 patients.^12^ Over the following 12 months, there were a further 86 cancers in test-negative individuals, corresponding to a sensitivity of 29.5% (95% confidence interval [CI] = 22% to 38%). However, only 9 (10.4%) of the 86 interval cancers were stages III or IV, and 38 were screen detected with a different screening test.^12^

At the end of 2020, we felt that the performance characteristics estimated from retrospective clinical studies justified evaluation of clinical utility in a prospective RCT. Modeling suggested that the impact of screening on prevention of late-stage cancer and reduction in cancer mortality could be substantial,^13^ however, we had no empirical evidence that there would be any clinical benefits from annual screening. NHS-Galleri was designed to provide that evidence.

Alternative designs were briefly considered but quickly rejected. A single-arm prospective trial (such as PATHFINDER^14^) has a role in demonstrating that testing asymptomatic individuals can detect cancer and to empirically validate the positive predictive value in a screening population. Although it also provides data on the stage distribution of screen-detected cancers, this would not be sufficient to demonstrate clinical benefit of screen detection, nor would it be sufficient to justify a screening program. Similarly, although it might be possible to demonstrate impact on cancer mortality through a well-designed large-scale pilot implementation, we did not feel that the evidence justified such a pilot.

#### Sensitivity

Although it is desirable for a screening test to have high sensitivity for early stage disease, it is not necessary. Screening with the guaiac fecal occult blood test has reduced colorectal cancer mortality in many countries despite a sensitivity of only 40%-70%. By comparison, the sensitivity of Galleri, in a retrospective study, to detect colorectal cancer was 82%: 43% for stage I and 85% for stage II.^10^

A single-cancer test with 100% sensitivity for the targeted cancer would be unlikely to have an all-cancer sensitivity of more than 10%-30% in a screening population. For instance, in the United Kingdom, breast cancer accounts for approximately 30% and bowel cancer for approximately 11% of all cancers; and 32% of all cancers diagnosed in the National Lung Screening Trial, a trial targeting individuals with high lung cancer risk, were lung cancer.^15^

The usefulness of cancer screening depends on its benefits and harms. The higher the sensitivity to potentially fatal cancers the better, but the potential impact of population screening with a multicancer early detection test of modest sensitivity may still be substantial. Using published site- and stage-specific sensitivities for Galleri, and the site- and stage-specific fatality, modeling predicts that annual screening could, over the long-term, prevent 17% of cancer deaths in screened individuals^16^; 17% was the smallest modeled reduction from all the scenarios considered and included allowance for the stage-specific hazard ratio of a screen-detectable cancer being 3 times that in a cancer that is not screen detectable. It does, however, apply only to those who comply with annual screening. Such a reduction is by no means certain, but the potential necessitates generation of high-quality RCT evidence to determine whether it can be achieved. A more recent microsimulation model of 14 solid tumor cancer types predicted an 18% reduction in cancer mortality over a 10-year horizon in the general US population.^17^

A 17% reduction in cancer deaths would be transformative. By comparison, initiatives based on accelerating diagnosis in symptomatic patients have had, at most, modest impact on mortality. Trends in cancer mortality in the United Kingdom between 1993 and 2018^18^ provide no indication of an accelerated decline following the introduction of 2-week wait referrals for suspected cancer in 2000.

The modeled 17% reduction in cancer mortality is after several years of annual screening. We anticipate a much smaller effect within a year of the first prevalent screen because most cancers that result in death within a year of random assignment will have already been advanced at the time of the first blood draw, and screening may have been offered too late to make a difference.

The lack of sensitivity of Galleri to nonaggressive cancers such as thyroid, low-grade prostate, and screen-detected estrogen receptor–positive breast^10^ means, together with the above mentioned correlates of ctDNA shedding (tumor burden, proliferation, and genome instability), that we are less concerned about the potential for overdiagnosis. Although there could be some overdiagnosis of hematological cancers. NHS-Galleri will study overdiagnosis. Four years after enrollment of the last participant, we will test the stored baseline sample from control-arm participants who have been diagnosed with cancer. We will then compare the cumulative incidence of baseline test-positive cancers between the arms. We anticipate an initial excess in the screening arm, because of earlier diagnosis, which will decrease over time. By restricting analysis to the small proportion of participants who may have been overdiagnosed from their baseline screen, we greatly improve the power to study overdiagnosis.^19^

#### Specificity

Some commentators have questioned the practicality of extensive investigation of positive Galleri tests. With annual screening from age 50 to 77 years (close to 100% uptake and 99.5% specificity), there would be approximately 100 000 individuals with a false-positive screen in England each year.^20^ However, in 2022-2023, there were 2.98 million urgent referrals for suspected cancer, an increase of 147 960 from the previous year, and 94% of these did not lead to cancer diagnosis^21^; 100 000 additional negative investigations, although substantial, is less than 4% of the current total. If such an increase were to result in 10% reduction in cancer mortality, it would be worthwhile.

Galleri’s specificity of 99.5%^10^ compares favorably with that of other screening tests. The specificity of the bowel screening fecal immunochemical test is approximately 94%,^22^ and the specificity of mammographic screening is 90%-98%. Equivalently, 12 of every 200 people screened by fecal immunochemical test have a false-positive test compared with 1 in every 200 with Galleri.

### Precautionary principle

A balance is needed between the precautionary principle (ie, screening should not be introduced until proven to cost-effectively do more good than harm) and guarding against the “perfect” becoming “the enemy of good” (ie, rejecting or delaying the introduction of a good screening program because the evidence, although strong, is not overwhelming). Historically, it has taken 10-15 years from publication of RCT evidence of a benefit to complete rollout of a cancer screening program. For instance, in 1996,^23^^,^^24^ 2 RCTs showed screening by fecal occult blood testing reduced mortality from colorectal cancer. A national bowel cancer screening program was introduced in 2006 and completed rollout in 2010.^25^ UK bowel screening was predicted to prevent approximately 2000 deaths per year.^26^ If screening had been safely and efficiently rolled out 10 years earlier, some 20 000 deaths would have been avoided. More needs to be done to accelerate national uptake of cost-effective screening. A first step would be to accelerate the generation of quality evidence.

A UK multicancer early detection screening program might involve collection of plasma from 5 to 18 million people each year, depending on age range and frequency of screening, and the management of 50 000-200 000 positive results. This will be a substantial undertaking. We should be thinking about that challenge now. There are many blood-based multicancer early detection tests being developed or evaluated for screening. If one is shown to substantially reduce cancer morbidity and mortality with little harm, we will want screening to be rolled out quickly and efficiently while ensuring that all sections of society benefit. Even a 2-year delay in implementation could result in an additional 28 000 cancer deaths in the United Kingdom alone.

An independent health economic analysis of the NHS-Galleri trial is planned. Cost-effectiveness is outside of the scope of this paper, but others have estimated an incremental cost-effectiveness ratio of $66 000 per quality-adjusted life-year (QALY) (range = $49 000-$116 000 depending on assumptions) based on annual screening with a test that costs $949.^27^ It seems likely that the cost of liquid biopsies will fall exponentially over the coming decade. With apologies to health economists, we offer this crude calculation as to what price per test multicancer screening might be cost-effective. We assume there is no impact on the cost of cancer treatment; others have estimated a saving of $5241 per person screened annually from age 50 to 79 years primarily based on a reduction in stage IV cancer, which is extremely expensive to treat in the United States,^27^ and a willingness to pay is $30 000 per QALY (noting that a threshold of $100 000/QALY is often used in North America). If, on average, screening those aged 50-79 years yielded 10 QALYs per cancer death avoided, then one would need to prevent 1 death for every 2000 screens (ie, 50 per 100 000) to make it affordable at $150 per test. Cancer mortality aged 55-84 years is approximately 800 per 100 000 people per year, so screening at $150 per test would need to prevent approximately 6.25% (50 of 800) cancer deaths. If annual screening prevented 17% of cancer deaths, the test could cost up to $400 and still meet the willingness-to-pay threshold. A more expensive test might only be used every 18 months or biennially or might be restricted to a narrower age group. Some will consider these speculations as premature and too rosy, but it is only through a trial that we will be able to better estimate the likely impact on QALYs.

### Trial design

There are 2 aspects of the NHS-Galleri design that are unusual. It is the first double-blinded cancer screening trial, and the primary endpoint is incidence of advanced stage cancer—most cancer screening trials use cancer-specific mortality.

#### Blinding

In drug trials, blinding is the gold standard. In NHS-Galleri, random assignment takes place after participants give their first blood sample; they are only unblinded if they are in the intervention arm and their test is positive. Thus, participants are invited to return for annual blood samples without knowing which arm they are in. Previous cancer screening trials have not attempted blinding either because of concerns about placebo tests or because participants randomly assigned to the control arm do not realize that they are part of a trial (Zelen design).

Nevertheless, NHS-Galleri has been criticized because it will not permit evaluation of change in behavior following a negative screen.^28^ However, it is not possible to estimate the impact of a negative routine screen on behavior from an RCT of an experimental screening test. In routine screening, participants are told that there is strong evidence that the benefits outweigh the harms and that a negative screen means low-risk not no-risk.^29^ By contrast, trial participants are told that the screening test may or may not work.

There are, however, advantages of collecting and storing blood from control-arm individuals. Thanks to blinding, random assignment can only influence mortality in those with a positive screen. At 5 years, we will test all stored samples from control participants who have died of cancer. By focusing on cancer deaths in those with a positive Galleri test, we have good power to study cancer mortality.^19^^,^^30^ There is no need to test all control samples because we aim to show a reduction in *cancer deaths with a positive sample* amongst all those randomized rather than *cancer deaths* amongst those with a positive sample.

#### Trial endpoint

Traditionally, trials of screening for invasive cancer use target-cancer mortality as the primary endpoint.^31^ Increasingly, people are concerned that using cancer-specific mortality as the only primary endpoint is too slow and too blunt an instrument.^32^ Although the primary endpoint of NHS Galleri is advanced stage cancer, cancer mortality will be reported 2 years later. This acknowledges that it is theoretically possible for a screening test to reduce late-stage cancers without impacting on cancer mortality. This could happen for a variety of reasons. (1) Screen-detected cancers could be “born to be bad”. That is, despite being diagnosed at an early stage, these cancers are highly aggressive and do not respond to treatment. (2) Earlier initiation of treatment for screen-detected cancers makes no difference—the patient will die on the same date whether diagnosed via screening or later via symptoms. This might arise if there is little difference in survival between early and late-stage cancers, due to either poor survival even of early stage cancer or excellent survival even of late-stage cancer.

The UK Collaborative Trial of Ovarian Cancer Screening,^33^ UK Flexible Sigmoidoscopy Trial,^34^ the Telemark trial of flexible sigmoidoscopy,^35^ and the lung component^36^ and ovarian component^37^ of the US Prostate, Lung, Colorectal and Ovarian trial each took at least 15 years to publish the impact of screening on cancer mortality. The National Lung Screening Trial took 9 years.^3^ With cutting-edge technologies, screening and treatment will have advanced over such a period, and a definitive answer regarding the trial intervention will no longer be relevant. Negative trials about chest X-rays for lung cancer screening tell us nothing about low-dose computed tomography. The trials of mammographic breast screening were mostly conducted when treatments for early and late breast cancers were different from those used today, and at all stages, 5- and 10-year relative survival were far worse than they are today. Thus, the mortality advantage of diagnosing a breast cancer at stage II rather than at stage III will be different than in the trials, and it is not possible to directly infer the mortality benefits of screening. The rapid pace of advances in liquid biopsies makes the case for accelerated research programs. Carrying out different studies in parallel—rather than in series—will help, but we also need to consider alternative endpoints that provide a good reflection of a meaningful outcome.

The reasons for use of stage III and IV cancers as the primary endpoint are as follows:

1. Modeling suggests that if Galleri screening reduces cancer mortality, it will work primarily by preventing cancers from progressing to stage III or IV.^38^
2. Meta-analyses^39^^,^^40^ of cancer screening trials show that the reduction in the incidence of advanced cancer in each trial is correlated with the reduction in cancer mortality in that trial. Although the authors of these 2 meta-analyses disagree on the interpretation of those findings, when combining results from RCTs of several types of cancer screening, there are only a few outliers to the general trend.^41^ Thus, the evidence to date is that a statistically significant reduction in all stage III and IV cancers will only be observed if screening causes a reduction in cancer mortality. There are concerns that these old trials were mostly based on imaging or protein biomarkers, whereas Galleri is looking for circulating tumor DNA. Because shedding of DNA is a hallmark of an aggressive cancer, there is concern that cancers screen detected by Galleri are born to be bad, and even those diagnosed at an early stage will be rapidly fatal. Although stage-for-stage cancers detectable by Galleri have a worse prognosis than those that are not detectable, there is no suggestion that their survival is any worse than observed in the Surveillance, Epidemiology, and End Results program as a whole.^42^
3. A trial powered to study cancer mortality would need to be substantially larger, require at least a further 2 years of follow-up after the last screen, and likely require an additional 2 rounds of screening. For instance, Hu, Prorok, and Katki^43^ propose a trial in 200 000 people lasting 7-9 years with 5 annual screens. By comparison, NHS-Galleri randomly assigned 140 000 individuals with 3 annual screens and will publish approximately 4 years after starting.
4. We will study the numbers who either die of cancer or are diagnosed with stage III and IV cancer in a sensitivity analysis. This endpoint might be called death-updated stage.^44^
5. The main outcome is advanced stage at 3 years. However, everyone will be followed at least until 5 years after the last participant was randomly assigned, at which point the trial will be analyzed in terms of cancer mortality.

If there is a statistically and clinically significant reduction in advanced stage, and if other safety and health-economic criteria are met, we anticipate there will be a pilot implementation of a Galleri-based screening program. Importantly, if there is a statistically significant reduction in advanced cancer at 3 years but no reduction in cancer mortality at 5 years (using the nested analysis), such a pilot should not lead automatically to a screening program. Rather one would need to synthesize the evidence from a variety of studies to determine whether it is the reduction in advanced stage cancer or the absence of reduction in cancer mortality that is the aberrant result. But if the results are consistent, by acting on the 3-year results, society will have gained 2 years, and many thousands of people will have been prevented from dying from cancer.

It has been suggested that waiting 2 years for the mortality outcomes would be sensible. We do not agree with this. What is gained by waiting for mortality? Whether or not we wait, we would want to run a large screening pilot. Such a pilot would be substantially cheaper per 10 000 individuals screened than an RCT. The safety, in terms of harms, will already be established from the RCT; there will be little additional evidence on harms from the additional follow-up because most harms from screening are almost instant. If screening has substantial harm, there will be no pilot. Pilots of cancer screening in the United Kingdom have not inevitably led to immediate rollout of that screening; the use of human papillomavirus testing to triage low-grade cytology is a case in point. If the pilot is well designed, it will provide considerable additional data on mortality. Had the trial been designed to study mortality as the primary outcome, we agree with Hu et al.^43^ that it would need to be 50% larger and to run for an additional 3-4 years—not simply an additional 2 years.

Provided the trial is not adversely affected by lack of adherence with annual testing, a negative result in terms of advanced stage will imply that, at best, the clinical benefit of annual screening with the Galleri test is modest. By showing in just over 4 years that annual screening with Galleri provides insufficient clinical benefit, we will limit investment in this one trial and allow the cancer screening community to move on and consider trials of newer, more sensitive multicancer early detection tests much sooner than had we waited for a trial powered for a mortality endpoint that would likely have taken a further 3 years.

## Conclusion

The only way to know whether regular screening with a multicancer early detection test can reduce cancer morbidity and mortality is through a well-designed and executed RCT. However, in our opinion, traditional approaches have not necessarily always served society well. Pragmatism demands that we consider new designs. NHS-Galleri is uniquely placed to provide early answers to important questions related to the benefits and harms of multicancer early detection screening, using innovative and robust study designs.

## Data Availability

No data were generated or analyzed for this manuscript.
